# *Sox9* mediates Notch1-induced mesenchymal features in lung adenocarcinoma

**DOI:** 10.18632/oncotarget.1970

**Published:** 2014-05-13

**Authors:** Kathleen M. Capaccione, Xuehui Hong, Katherine M. Morgan, Wenyu Liu, Michael J. Bishop, LianXin Liu, Elke Markert, Malik Deen, Christine Minerowicz, Joseph R. Bertino, Thaddeus Allen, Sharon R. Pine

**Affiliations:** ^1^ Department of Pharmacology, Rutgers Graduate School of Biomedical Science, Piscataway, New Jersey; ^2^ Rutgers Cancer Institute of New Jersey, New Brunswick, New Jersey; ^3^ Department of Surgery, The First Affiliated Hospital of Harbin Medical University, Harbin, China; ^4^ G.W. Hooper Research Foundation, University of California, San Francisco, CA; ^5^ Department of Medicine, Robert Wood Johnson Medical School, New Brunswick, New Jersey; ^6^ Department of Pathology, Robert Wood Johnson Medical School, New Brunswick, New Jersey

**Keywords:** Sox9, Notch1, TGF-β, lung cancer, EMT

## Abstract

*Sox9* has gained increasing importance both functionally and as a prognostic factor in cancer. We demonstrate a functional role for *Sox9* in inducing a mesenchymal phenotype in lung ADC. We show that *Sox9* mRNA and protein are overexpressed in lung ADC, particularly those with *KRAS* mutations. *Sox9* expression correlated with the Notch target gene *Hes1*, and numerous other Notch pathway components. We observed that *Sox9* is a potent inducer of lung cancer cell motility and invasion, and a negative regulator of E-cadherin, a key protein that is lost during epithelial-mesenchymal transition (EMT). Moreover, we show that Notch1 signaling directly regulates *Sox9* expression through a *SOX9* promoter binding site, independently of the *TGF-β* pathway, and that *Sox9* participates in Notch-1 induced cell motility, cell invasion, and loss of E-cadherin expression. Together, the results identify a new functional role for a Notch1-*Sox9* signaling axis in lung ADC that may explain the correlation of *Sox9* with tumor progression, higher tumor grade, and poor lung cancer survival. In addition to Notch and *TGF-β*, *Sox9* also acts downstream of *NF-κB* and *Wnt*/β-catenin signaling. Thus, *Sox9* could potentially act as a hub to mediate cross-talk among key oncogenic pathways in lung ADC. Targeting *Sox9* expression or transcriptional activity could potentially reduce resistance to targeted therapy for lung ADC caused by pathway redundancy.

## INTRODUCTION

*Sox9* is a member of the high-mobility group-box class DNA-binding protein family of transcription factors and plays a critical role in embryonic development, cell fate determination and lineage commitment. Specifically, *Sox9* orchestrates chondrogenesis, bone formation and testis development, among others [1,2]. Germline *SOX9* mutations cause campomelic dysplasia, a disorder characterized by numerous skeletal abnormalities and XY sex reversal [3]. Infants with mutant *SOX9* usually die due to respiratory distress shortly after birth [4]. This clinical finding likely reflects the role of *Sox9* in the developing lung. *Sox9* is highly expressed in the developing lung, and is required for branching morphogenesis in the lungs and for controlling alveolar epithelial progenitor cell proliferation [5-6].

Overexpression of *Sox9* is found in more than 50% of lung adenocarcinomas (ADCs), the most common histological lung cancer subtype, and is associated with a poor lung ADC survival [8]. Over the past 5 years, it has become increasingly clear that *Sox9* plays a major role across numerous human cancers. *Sox9* overexpression acts as an oncogene in several cancer types, including breast, colorectal, pancreatic, and prostate cancer and glioma [9-13], although it appears to act as a tumor suppressor in bladder cancer and melanoma [14,15]. In lung cancer, *Sox9* overexpression was recently shown to increase cell proliferation and xenograft tumor formation [8,16]. However, the functional role of *Sox9* in lung cancer has not been fully elucidated. Furthermore, the upstream oncogenic molecular pathways regulating *Sox9* overexpression in lung ADC have not been completely delineated.

The Notch signaling pathway regulates cell fate decisions in nearly every developing tissue and organ, and maintains adult tissue homeostasis. In humans, the Notch receptor is activated after binding to one of 5 Delta or Jagged ligands that are expressed on neighboring cells. Ligand binding initiates a series of cleavage events, and the final cleavage is carried out by the γ-secretase protease complex which frees the intracellular domain portion of Notch (ICD), allowing Notch ICD to translocate to the nucleus. Nuclear Notch ICD complexes with the transcriptional repressor *RBP-Jκ*, converting it into a transcriptional activator [17]. Notch ICD-induced effects on gene expression are tightly controlled by numerous post-translational modifications and protein co-factors. Several Notch target genes are nearly universally activated across many cell types and developmental processes, such as Hes and Hey, while many other Notch target genes are highly cell-type and context specific [18,19].

Notch1 is a putative oncogene that is aberrantly over-activated in over one-third of lung ADCs through gain-of-function mutations (10%) or altered expression of Notch receptors and/or suppressors (30%) [20]. Notch1 is required for tumor initiation in the *KRAS*-driven lung ADC mouse model [21]. Moreover, overexpression of constitutively activated Notch1 directly participates in lung carcinogenesis; it induces lung adenomas in mice which progress to ADCs in cooperation with Myc overexpression [22]. Aberrant Notch1 signaling contributes to acquired resistance to EGFR inhibitors, the most widely used targeted therapy for NSCLC [23]. Functionally, Notch increases lung cancer stem cell (CSC) self-renewal and promotes epithelial-mesenchymal transition (EMT) [23,24], reviewed in [25], although the downstream mechanisms mediating these effects have not been fully defined. Few studies have identified specific target genes of oncogenic Notch1 in lung ADC, but those validated thus far include Survivin and *IGF1R* [26,27]. Because of the exquisite specificity of cell-type and context specific effects of Notch signaling, it is necessary to explore Notch target genes in lung cancer in order to elucidate the genes mediating Notch-induced EMT within this disease.

*Sox9* is a well-studied downstream effector of the Notch pathway in murine tissues, especially during development [28-34], through *RBP-Jκ* binding sites located at positions +40 bp and −325 bp relative to the *SOX9* transcriptional start site [35]. However, these binding sites are not conserved in humans. *Sox9* expression correlates with Notch signaling in humans [36], but there has been a lack of direct evidence that Notch-induced modulation of *Sox9* expression is evolutionarily conserved in humans or that *Sox9* is a Notch target in lung cancer. Loss of the *TGF-β* adaptor *β2SP* induces *TGF-β* signaling, resulting in both Notch activation and *SOX9* expression in esophageal ADC [37]; although, the specific role of Notch in regulating *Sox9* expression was not fully delineated. In this study, we show that the Notch1-*Sox9* signaling axis is evolutionarily conserved through a novel *RBP-Jκ* binding site at -10 bp relative to the *SOX9* promoter and that *Sox9* expression is regulated by Notch1 in lung ADC independent of *TGF-β* signaling. Further, we demonstrate that *Sox9* mediates Notch1-induced cell motility, invasion, morphological changes, and suppression of E-cadherin, all features of a mesenchymal phenotype.

## RESULTS

### *Sox9* expression is elevated in KRAS-mutant lung ADC

**Figure 1:**
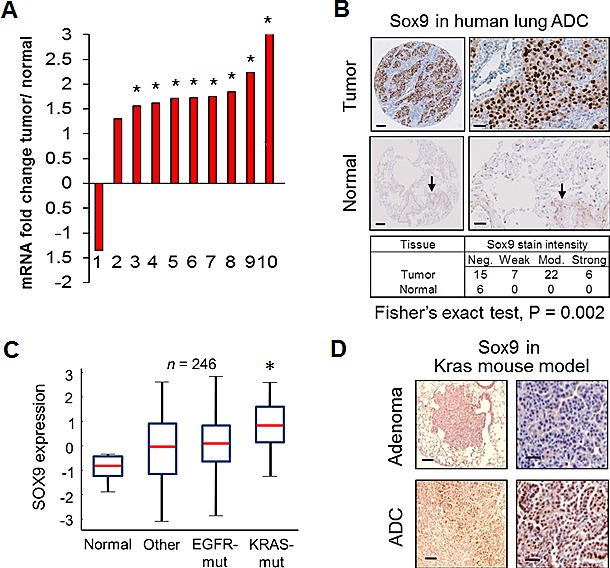
*Sox9* expression in lung adenocarcinoma (A) Fold change in log2 median-centered intensity of mRNA expression levels in lung ADC samples, as compared to normal lung tissue samples, in publicly available datasets. Fold change in tumors is relative to 1 for normal tissues; *, P < 0.05. (B) Surgical lung ADC samples were immunohistochemically stained for *Sox9*. Representative staining is shown in human lung ADC and normal adjacent tissue samples. Quantified scores of staining intensity are shown. Arrows indicate light staining in the vascular endothelium. Scale bar for left panel, 100 μm and for right panel, 25 μm. (C) Normalized *SOX9* mRNA expression levels from human lung ADC samples categorized by genetic subgroups were compared to their respective sets of normal adjacent tissues; other, wildtype for both *KRAS* and *EGFR*; *, P < 0.05. (D) Representative immunohistochemical staining (N = 4 mice) of lung tissue sections for *Sox9* is shown from the LSL-K-rasG12D lung ADC mouse model. Scale bar for left panel, 200 μm and for right panel, 50 μm.

An analysis of publicly available datasets [38-47] on Oncomine (www.oncomine.com) revealed an upregulation of *Sox9* mRNA in the majority of lung ADC datasets. Out of 10 available datasets comprising a total of 1,021 samples, 8 datasets with 762 samples showed statistically significant upregulation of *Sox9* in lung ADC tumor tissue in comparison to normal adjacent tissue (Fig. 1A). We screened a panel of 14 lung ADC cell lines, and observed detectable levels of *Sox9* protein in 10 cell lines. Low *Sox9* expression was found in immortalized bronchial epithelial Beas2b cells (Supplementary Fig. S1). We further investigated *Sox9* expression at the protein level in 50 human lung ADC surgical samples and paired NATs. *Sox9* protein levels were significantly higher in tumors than in NAT (Fig. 1B). *Sox9* protein was predominantly nuclear and overexpressed in 70% of human ADCs. In contrast, normal adjacent lung tissue was scored negative, though vascular endothelial cells showed weak positive nuclear and cytoplasmic staining (Fig. 1B), and, when present, bronchial epithelial cells showed moderate cytoplasmic staining with focal nuclear staining.

To further investigate *Sox9* overexpression in lung ADC, we queried data with annotated mutational information and analyzed *Sox9* expression within lung cancer genetic subgroups, including EGFR and *KRAS* mutations. *Sox9* mRNA was significantly higher in lung ADCs with *KRAS* mutations compared to NAT. EGFR mutant tumors had a trend of higher *Sox9* expression than in NAT, but it was not statistically significant (Fig. 1C). These data are consistent with the report that *Sox9* overexpression is required for oncogenic *Kras*-induced pancreatic intraepithelial neoplasia [48]. To investigate if high *Sox9* expression is conserved in a GEMM of lung ADC, we stained lung adenoma and ADC sections from the LSL-K-rasG12D lung ADC mouse model for *Sox9* and observed *Sox9* protein expression to be mostly absent from normal lung and adenoma tissue, but highly expressed in ADC tumors (Fig. 1D). Expression was not uniform in the tumors and was restricted to the more malignant-appearing outgrowths.

### *Sox9* is a Notch-responsive gene during murine lung carcinogenesis

**Figure 2:**
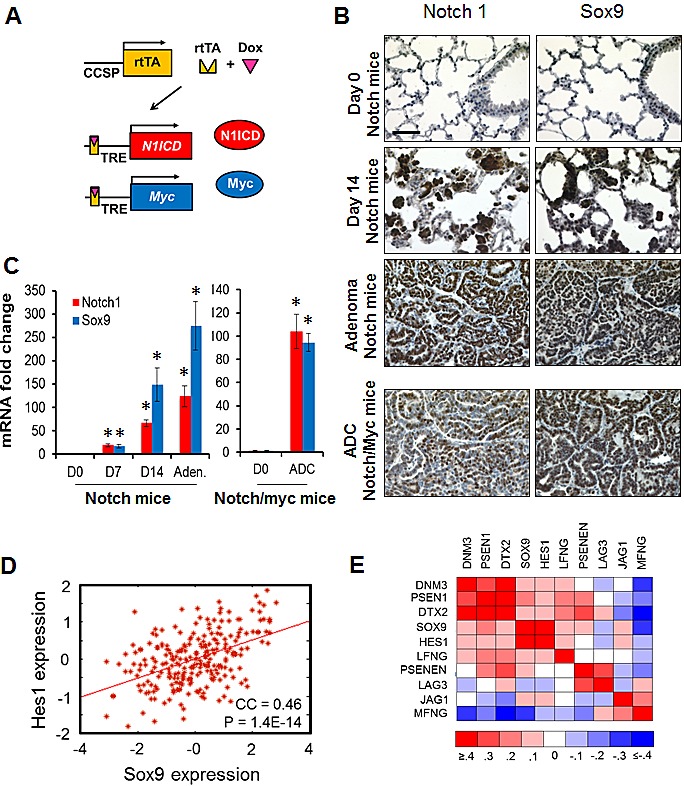
*Sox9* is downstream of Notch1 during murine carcinogenesis and correlates with the Notch pathway (A) Schematic depicting how Myc and Notch1 transgenes were conditionally expressed in lung epithelial cells with doxycycline (Dox) treatment. (B) Lung tissue sections from the Notch1 (Day 0, Day 14 and adenoma) or Notch1/Myc (Adenocarcinoma) lung cancer mouse models were immunohistochemically stained for *Sox9* and Notch1 expression. Scale bar, 50 μm; all pictures are the same magnification. (C) *SOX9* mRNA levels were assayed in a set of mouse lung tissues from the indicated conditions (N=3 mice each). Mean fold changes with standard deviations, compared to no doxycycline treatment, are shown; *, P < 0.05. (D) Linear regression of normalized *SOX9* and *HES1* mRNA levels [41]. (E) Heat map of Pearson correlation coefficients of mRNA gene expression across *Sox9* and genes that modulate the Notch pathway. Raw data and P values are detailed in Supplementary Table 1.

Notch1 was recently shown to be required for the LSL-K-rasG12D lung ADC GEMM [21]. We analyzed if *Sox9* is a downstream effector of Notch1 during lung carcinogenesis. Notch1 overexpression in the mouse lung alveolar epithelium, driven by the Clara cell specific protein (CCSP) promoter, leads to pulmonary adenomas that progress to ADCs when crossed with conditional Myc overexpression [22]. We tested these conditional GEMM of lung ADC to determine if *Sox9* expression is an early event downstream of Notch1 signaling (Fig. 2A). As previously reported, within 14 days of doxycycline induced *N1ICD* expression, hyperplastic regions were clearly discernable in stained lung sections (Fig. 2B) [22]. These hyperplastic regions progressed to adenomas with papillary histology in doxycycline-fed Notch1 mice. Immunohistochemical staining for Notch1 demonstrated high expression levels in the hyperplastic and adenomatous regions. Similarly, *Sox9* protein was substantially upregulated in the hyperplastic and adenomatous regions (Fig. 2B). Moreover, multifocal ADCs developed in mice conditionally expressing both *N1ICD* and Myc driven by the CCSP promoter when induced by doxycycline. Notch protein was highly expressed in this model, and *Sox9* displayed a similar pattern (Fig. 2B). To test if enhanced *Sox9* transcription was responsible for the increased *Sox9* protein levels in these mouse models, we assessed *Sox9* mRNA expression and found an 18-fold increase in lung tissue as early as 7 days after Notch1 induction (Fig. 2C). Levels continued to increase over time through to adenoma formation, and also in the ADC samples from the Notch1-Myc mice (Fig. 2C). These data indicate that *Sox9* is responsive to Notch signaling as an early event during Notch1-induced lung carcinogenesis *in vivo*.

To determine if *Sox9* expression correlates with Notch signaling in human lung ADC, we performed gene co-expression analyses across several datasets. There was a signficant correlation between *Sox9* and *Hes1* expression, a common Notch-target gene, in 3 of 3 examined studies (Fig. 2D and Supplementary Fig. S2). Both *Hes1* and *Sox9* positively correlated with the catalytic enzyme that cleaves Notch PS

EN1, the Notch ligand JAG1, and positive modulators of Notch signaling, DTX2 and LFNG. *Hes1* and *Sox9* both negatively correlated with MFNG, which inhibits ligand-dependent Notch activation (Fig. 2E and Supplementary Table S1).

### Sox9 is responsive to Notch1 signaling in human lung adenocarcinoma

**Figure 3:**
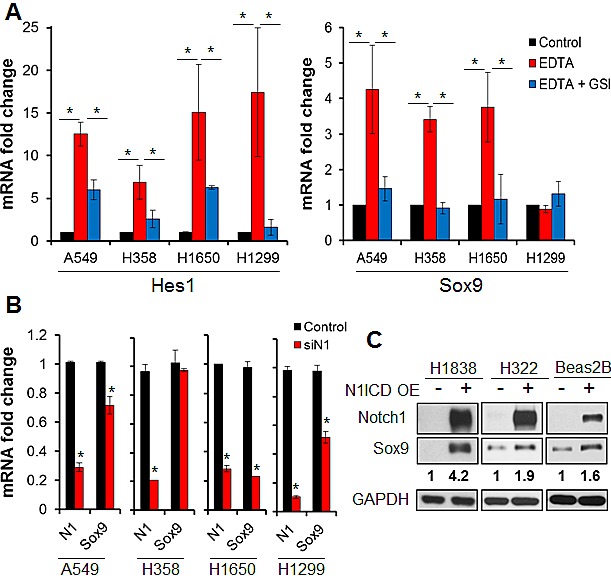
*Sox9* is downstream of Notch signaling in human lung cancer (A) Mean mRNA levels and standard deviations of *HES1* and *SOX9* assessed by quantitative RT-PCR are shown in untreated lung ADC cells (Control), after EDTA-induced Notch activation (EDTA), and after Notch activation was blocked by a gamma secretase inhibitor (EDTA + GSI); *, P < 0.05. (B) mean mRNA fold change and standard deviations of Notch1 and *Sox9* are shown in lung adenocarcinoma cells 72 hours after transient transfection with siRNA against Notch1 as compared to transfection with a scrambled siRNA (Control); *, P < 0.05. (C) Expression of Notch1 and *Sox9* were analyzed by western blot in lung ADC and immortalized bronchial epithelial cells 48 hours after transfection with vector or Notch1 intracellular domain. Quantification of *Sox9* by densitometry, normalized to *GAPDH* protein levels, is shown.

Because *Sox9* was a reported target gene of the Notch pathway during murine development and we found a significant correlation between the Notch pathway and *Sox9* expression at the mRNA level across microarray datasets of lung ADC samples, we set out to determine if *Sox9* is a key gene downstream of Notch in lung ADC. We treated lung ADC cells with EDTA, known to induce Notch activation by chelating calcium [49]. EDTA-induced Notch activation consistently upregulated expression of *Hes1* at least 5-fold at both the RNA and protein levels (Fig. 3A and Supplementary Fig. S3). We observed dose-dependent inhibition of Notch by the gamma-secretase inhibitor (GSI) RO4929097 (Supplementary Fig. S4). The GSI substantially blocked EDTA-induced *Hes1* expression (Fig. 3A, left panel). Similarly, *Sox9* expression was significantly increased after EDTA-induced Notch activation in 3 of 4 lung ADC cell lines examined, and *Sox9* upregulation was significantly eliminated in the presence of the GSI (Fig. 3A, right panel), suggesting that *Sox9* is highly responsive to Notch signaling in lung ADC. The intensities of upregulated *Sox9* and *Hes1* expression induced by Notch signaling were different, as is typical of Notch-target genes [28,50].

We next set out to determine if *Sox9* is downstream of Notch1 or Notch3, the two Notch receptors implicated in lung ADC [20,24,26]. We knocked down Notch1 expression in H1299 cells, which had high basal *Sox9* protein levels among the screened cell lines, and *Sox9* mRNA and protein expression were repressed (Fig. 3B, Supplementary Fig. S5A). Similar results were observed in A549 and H1650 cells, but not in H358 cells (Fig. 3B), suggesting that other upstream pathways might also drive *Sox9* expression in lung cancer. Conversely, knockdown of Notch3 failed to decrease *Sox9* expression in any of the 4 cell lines tested. However, in two cell lines, Notch3 silencing increased *Sox9* expression, concomitant with an increase in Notch1 mRNA caused by Notch3 knockdown (Supplementary Fig. S5B and C). This was likely due to a compensatory increase in Notch1 secondary to decreased Notch3 signaling, a known Notch1 antagonist [51]. Overexpression of the active form of Notch1 (*N1ICD*) induced *Sox9* expression in lung ADC cell lines and immortalized bronchial epithelial cells that had low basal *Sox9* levels (Fig. 3C). These data confirmed that Notch1 signaling influences *Sox9* expression in human lung ADC.

### Notch1 directly regulates Sox9 gene expression

**Figure 4:**
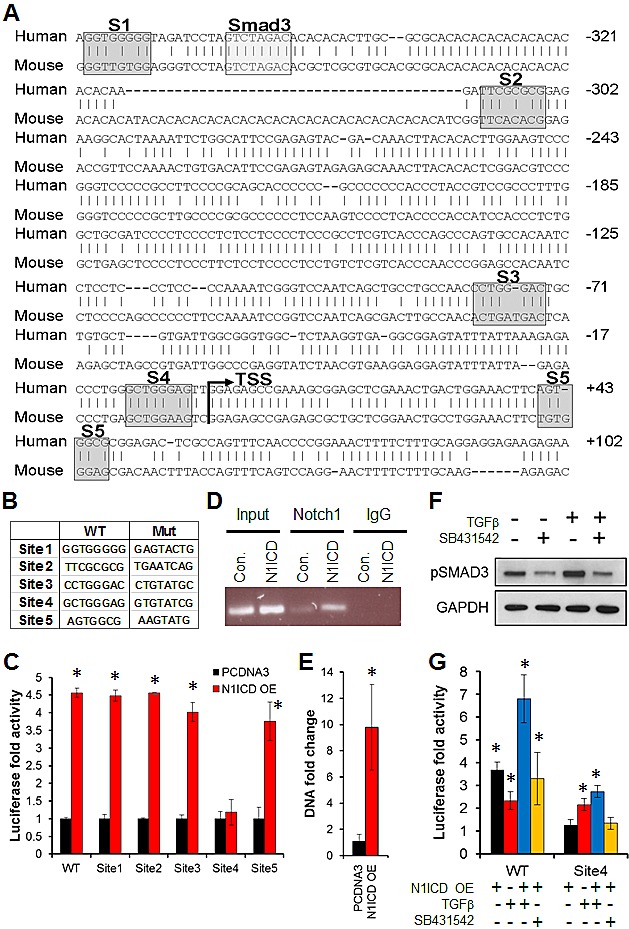
*Sox9* is a Notch1 target gene in lung adenocarcinoma (A) Alignment of the human and mouse *SOX9* promoter sequences is shown, with indicated putative *RBP-Jκ* binding Sites 1 - 5 and Smad3 binding element, relative to the *SOX9* transcriptional start site (TSS). Sites 1, 2, and 5 were previously reported (See text), and we identified Sites 3 and 4 through LASAGNA, (B) Wild-type and mutated primer sequences used for site-directed mutagenesis of Sites 1 - 5 are shown. (C) Normalized luciferase fold activity (to Renilla luciferase) and standard deviations in cells co-transfected with Notch1 intracellular domain (*N1ICD* OE) and a luciferase reporter driven by a 1 kb fragment of the wild-type or mutated *SOX9* promoter in sites 1 – 5, compared to the vector control (PCDNA3); *, P < 0.05. (D) ChIP was performed using either a Notch1 or IgG control antibody on cells transfected with *N1ICD* or a control vector (PCDNA3), and the *SOX9* promoter was amplified by PCR, using primers that were designed to amplify Site 4. (E) Quantitative PCR was performed after ChIP; *, P < 0.05. (F) pSmad3 was analyzed by western blot after treatment with vehicle controls, recombinant human *TGF-β* or the *TGF-β* inhibitor, SB431542. (G) Normalized luciferase fold activity (to Renilla luciferase) is shown in cells transfected with *N1ICD* and a luciferase reporter driven by a 1 kb fragment of the wild-type or mutated *SOX9* promoter at Site 4, and treatment with *TGF-β* or SB431542, each compared to its empty vector control, which was set to 1 (Not shown); *, P < 0.05.

Recently, two *RBP-Jκ* binding sites in the murine *SOX9* promoter were identified at approximately −325 bp (S2) and +40 bp (S5) relative to the transcriptional start site (Fig. 4A) [35,52]. Alignment of the mouse and human *Sox9* promoters revealed that these mouse *RBP-Jκ* binding sites are not conserved in humans (Fig. 4A). We queried −950 to +50 bp relative to the human transcriptional start site using the LASAGNA algorithm [53] and identified two novel *RBP-Jκ* binding motifs with potential high affinity located at positions −81 bp (S3) and −10 bp (S4) (Fig. 4A). To test whether direct binding of *N1ICD* to these *RBP-Jκ* binding sites in the human *Sox9* promoter is necessary for induction of *Sox9* expression in human NSCLC, we used a luciferase reporter under the control of a 1 kb-fragment of the *Sox9* promoter, transiently expressed in the *Sox9*-low H1838 cells. Activity of the wild-type reporter was induced 4.5-fold, when co-transfected with *N1ICD* (Fig. 4C). We mutated the four putative *RBP-Jκ* binding sites, as well as one additional site (Site 1) proposed by Song et al. [37] to cooperate with the Smad3 SBE (Smad binding element) upstream of *Sox9* in esophageal adenocarcinoma (Fig. 4B). Only mutation of Site 4, that we identified by LASAGNA, abolished activation of the luciferase reporter activity by *N1ICD*, showing that this −10 bp site is, indeed, a functional site responsible for the induction of *Sox9* by Notch1 in human lung ADC (Fig. 4C). Consistent with these data, we performed ChIP-QPCR assays on H1838 cells transiently transfected with *N1ICD*, using primers designed to amplify Site 4, and found that *N1ICD* physically associated with the *RBP-Jκ* binding site in the human *Sox9* promoter at or near the −10 bp site (Fig. 4D and E). Taken together, these results establish *Sox9* as a direct transcriptional Notch1 target in human lung ADC.

Because Smad3 was recently shown to interact with Notch1 in esophageal adenocarcinoma cells after treatment with *TGF-β*, we tested if *N1ICD* binding to the *Sox9* promoter *RBP-Jκ* site was dependent on *TGF-β* signaling in lung ADC. *TGF-β* increased Smad3 phosphorylation (Fig. 4F) and significantly induced *Sox9* reporter luciferase activity in both the wild-type and mutated Site 4 promoters by 2-fold (Fig. 4G). Combined *TGF-β* treatment with transient overexpression of N1ICD resulted in an additive increase in luciferase activity, compared to either one individually, indicating that *Sox9*-promoter driven gene expression does not require cooperativity of *N1ICD* and *TGF-β* in lung ADC. Further, *TGF-β* treatment induced an additive increase in the basal luciferase activity even with a mutation at Site 4Inhibition of *TGF-β* signaling by SB431542 abolished *TGF-β*-induced Smad3 phosphorylation (Fig. 4F) but had no effect on *N1ICD*-induced luciferase activity (Fig. 4G). These data confirm that Notch1 and *TGF-β* induce *Sox9* expression independently in lung ADC.

### Sox9 mediates Notch1-induced cell motility and suppression of epithelial-like cellular morphology

To determine if *Sox9* affects cell motility, we carried out *in vitro* wound-healing assays with H1299 and A549 cells. Knockdown of either Notch1 or *Sox9* significantly reduced migration, as compared to cells transfected with a scrambled siRNA sequence. Overexpression of *Sox9* rescued the reduced cell motility caused by loss of Notch1 signaling, in both H1299 (Fig. 5A and B) and A549 cells (Supplementary Fig. S6A). We then tested if silencing *Sox9* could block Notch-enhanced migration. Transient overexpression of activated Notch1 increased A549 cell motility, though not significantly. However, silencing of *Sox9* expression reversed the increased cell motility caused by activated Notch1, down to similar levels seen with silenced Notch1 (Supplementary Fig. S6B). These data demonstrate that *Sox9* at least partly mediates Notch1-induced cell migration.

**Figure:**
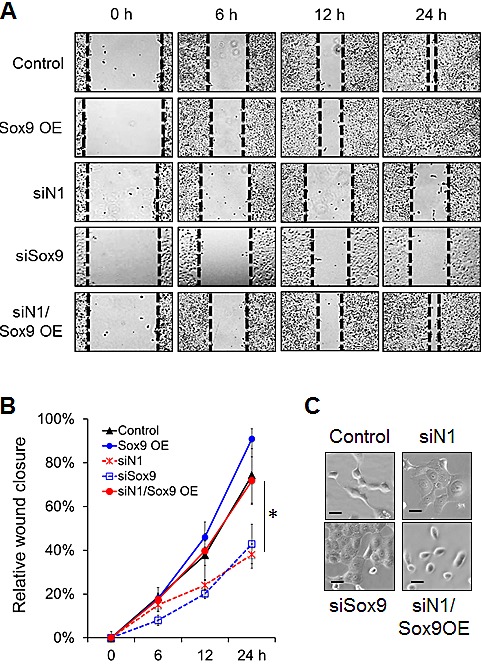
Figure 5: *Sox9* mediates Notch1-induced cell migration and morphological changes (A) Migration ability of lung adenocarcinoma cells was examined with a wound healing assay starting at 48 hours after transient knockdown of Notch1 or *Sox9*, or overexpression (OE) of *Sox9*. The control sample was transfected with both a scrambled siRNA sequence and empty vector. Representative images are shown at 0, 6, 12 and 24 hours. Black dotted lines indicate the wound edge closure of monolayer cells. (B) Mean migration distance of the wound edge, and standard error of the mean, is shown in all treatment compared to cells transfected with both a scrambled siRNA sequence and empty vector (Control); *, P < 0.05. (C) Representative brightfield images of lung ADC cells 72 hours after knockdown of Notch1 or *Sox9*, or *Sox9* overexpression, compared to cells transfected with both a scrambled siRNA sequence and empty vector (Control). Scale bar, 10μm.

H1299 cells are characterized by a mesenchymal gene expression signature and spindle-shaped morphology with few cell-cell contacts. Compared with control cells, H1299 cells with knocked down Notch1 or *Sox9* expression underwent profound morphologic changes, which included a larger, flattened phenotype and tighter, more numerous cell-cell contacts. When *Sox9* was overexpressed in cells with knocked down Notch1, the mesenchymal appearance of the cells was rescued, with characteristic spindle-like shape and loss of tight cell-cell contacts (Fig. 5C).

### Sox9 mediates Notch1-induced cell invasion and loss of epithelial marker expression

**Figure 6:**
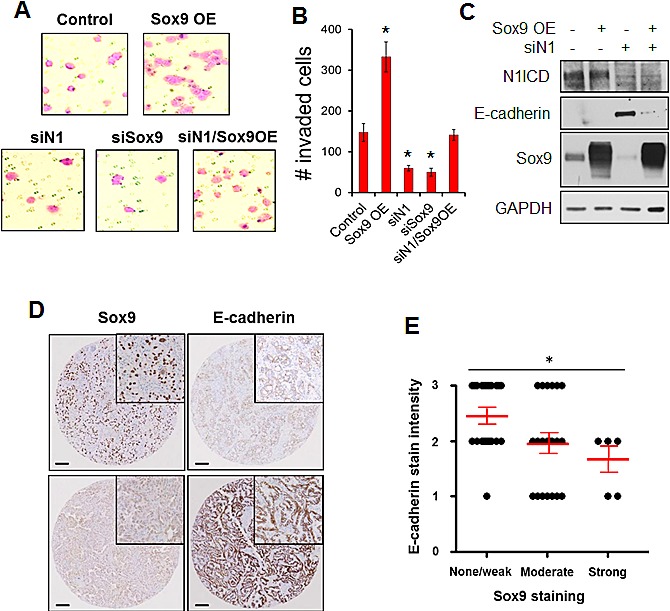
*Sox9* mediates Notch1-induced cell invasion and expression of E-cadherin (A) invasion assays were performed on H1299 lung ADC cells 48 hours after transient knockdown of Notch1 or *Sox9*, or overexpression (OE) of *Sox9* compared to cells transfected with both a scrambled siRNA sequence and empty vector (Control). Representative images show crystal violet-stained invaded cells. (B) Total number of invaded cells from experiments in A were quantified and compared to the control samples; *, P < 0.05. (C) Intracellular Notch1 (*N1ICD*), E-cadherin and *Sox9* were analyzed by western blot 72 hours after transient knockdown of Notch1 or overexpression of *Sox9*, or transfection with a scrambled siRNA sequence and empty vector, in H1299 cells. (D) Lung ADC tumors were immunohistochemically stained for *Sox9* and E-cadherin. Representative staining is shown in human lung adenocarcinoma samples with high (upper panel) or low (lower panel) *Sox9* staining. Scale bar, 100 μm. (E) Quantified *Sox9* and E-cadherin immunohistochemical stain intensity scores were graphed; *, P < 0.05.

Because our data suggested that *Sox9* mediates the adoption of a mesenchymal phenotype induced by Notch1, we analyzed cell invasion, a hallmark of mesenchymal cells. Transient Notch1 or *Sox9* knockdown significantly reduced invasion of H1299 cells through Matrigel-coated Transwell inserts, and transient overexpression of *Sox9* rescued the cells' invasive ability (Fig. 6A and B).

The cell-cell adhesion molecule E-cadherin is a major determinant of epithelial features. In line with the morphological changes, E-cadherin expression was substantially increased after knockdown of Notch1 expression in H1299 cells. Overexpression of *Sox9* substantially, though not completely, blocked the upregulation of E-cadherin expression caused by knock-down of Notch1 (Fig. 6C and Supplementary Fig. S6C). Similarly, in A549 cells, repression of Sox9 upregulated E-cadherin protein expression, even in the presence of activated Notch1 ICD overexpression (Supplementary Fig. S6D). Knockdown of Notch1 significantly reduced expression of *MMP9*, Slug and Snail, however, overexpression of *Sox9* did not rescue expression of these genes after Notch1 knockdown. Furthermore, modulation of *Sox9* expression did not affect mRNA levels of Vimentin, *CEACAM1*, Twist, *Zeb1* or *Zeb2* (Data not shown). To establish the correlation between *Sox9* and E-cadherin expression *in vivo*, we stained the human lung ADC tumors for E-cadherin. We found a significant inverse correlation between E-cadherin and *Sox9* staining intensities (Fig. 6D and E). Together, these findings suggest that *Sox9* may be a gatekeeper partly mediating Notch's role in EMT by inducing loss of epithelial features, blocking E-cadherin expression, and increasing cell motility and invasion.

## DISCUSSION

*Sox9* is a developmental gene required for lineage commitment in numerous tissues and organs, including the lung. It is well-accepted that genes regulating development also play critical roles in cancer development and progression, and thus it is not surprising that *Sox9* expression is dysregulated in cancer. *Sox9* is overexpressed in several tumor types and associated with poor survival [16,54,55]. Upregulation of *Sox9* expression was reported to increase cell proliferation [9,11,16], and induce invasion in pancreatic, prostate, and urothelial cancer [12,56,57]. *Sox9* cooperates with Slug to induce EMT in mammary stem cells, and tumor progression in breast cancer [10]. These reports in total indicate the importance of examining the pathways inducing *Sox9* dysregulation and to fully delineate the mechanisms through which *Sox9* participates in lung carcinogenesis and tumor progression.

We demonstrate that *Sox9* is overexpressed at the mRNA level across the majority of publicly available datasets and at the protein level in 50 primary lung ADC tumors, consistent with previous reports [16,55]. We show that *Sox9* is overexpressed within the lung ADC subgroup that harbors *KRAS* mutations, and confirm that *Sox9* is overexpressed within the more malignant portions of tumor from the LSL-K-rasG12D lung ADC mouse model [9]. The Notch pathway cooperates with *Kras* in development of murine pancreatic intraepithelial neoplasia (PanIN) [58] and lung ADC [21]. Furthermore, *Sox9* overexpression induces acinar-to-ductal reprogramming and cooperates with *Kras* during PanIN formation [48].

In *KRAS*-mutant lung ADCs, *Sox9* overexpression may be secondary event associated with tumor progression [9], rather than directly downstream of the *Kras* pathway. Conversely, in the murine model of Notch1-induced lung cancer, *Sox9* is overexpressed as early as 7 days after induction of Notch1 overexpression in the alveolar epithelium, specifically confined to the alveolar hyperplastic regions, and thus is likely to be directly downstream of Notch. We propose that *Sox9* participates as a primary downstream Notch effector early in Notch1-induced carcinogenesis, but as a secondary event during *KRAS*-mutant lung carcinogenesis, and both *Kras* and Notch contribute significantly to lung tumor formation. Further studies are needed to determine if, 1) *Sox9* overexpression is required for tumor formation in the *Kras* and Notch1 lung cancer mouse models, 2) if *Sox9* acts downstream of *Kras* or Notch in the *Kras* lung cancer mouse model, and 3) if *Sox9* alone can drive the development of premalignant or malignant lesions.

Aberrantly activated Notch signaling is associated with poor survival, CSCs, EMT, and chemoresistance in lung cancer [23,24,59,60]; however, very little is known about the downstream genes mediating the effects of Notch in lung ADC [26,27]. We show that *Sox9* is not only a direct target of Notch1 in lung ADC but also functionally mediates Notch1-induced cell motility, invasion, and mesenchymal-like characteristics. While we observed that *Sox9* overexpression decreased E-cadherin, the mode by which this happens is unclear. A search of 5 kb upstream of the E-cadherin transcription start site revealed no *Sox9* binding sites. Future studies are needed to investigate the mechanisms by which *Sox9* regulates E-cadherin expression. Work is also needed to examine if *Sox9* mediates Notch-induced chemo- and radio-resistance, as well as CSC self-renewal.

This study has important clinical implications since Notch signaling is aberrantly over-activated not only in 40% of lung ADCs but across numerous tumor types [17]. Gamma secretase inhibitors that inhibit Notch activation, and monoclonal antibodies are in clinical trials, but they are largely ineffective due to dose-limiting toxicities or development of resistance [61-63]. Clearly, novel methods to target the tumorigenic effects of Notch while minimizing toxicities are necessary. The results of this study could be an impetus for investigations for inhibiting *Sox9* transcriptional activity or expression.

## MATERIALS AND METHODS

### Cell culture and drug treatments

Human bronchial epithelial Beas2b cells were a kind gift of Dr. John Langenfeld, A549, H1299, H358, H1650, H322, H441, H1975, H838, H1838, H23, and H1755 cell lines were obtained from the American Type Culture Collection (ATCC), and Sk-Lu-1, H2030, and HCC-44 cell lines were a gift from Bristol Myers Squibb (Princeton, NJ). Cells were cultured in media as recommended by ATCC and maintained at 37° with 5% CO_2_ and ambient O_2_. For EDTA-induced Notch activation, cells were exposed to 1 mM EDTA or culture media for 30 min, then placed back in culture media for 1 hour. For inhibition of Notch activation, cells were treated with 1 μM RO4929097 or DMSO for 48 hours. Human recombinant *TGF-β* (PeproTech) was used at a concentration of 10 ng/ml for 48 hours. *TGF-β* inhibitor S431542 (Sigma) was used at a concentration of 10 μM for 48 hours.

### Human patient samples

Formalin-fixed paraffin embedded tissue specimens were obtained through the Tissue Retrieval Service, Rutgers Cancer Institute of New Jersey, from patients who underwent surgery at Robert Wood Johnson University Hospital. This study was approved by the Institutional Review Board of Rutgers Robert Wood Johnson Medical School.

### Genetically engineered mouse models (GEMM)

Animal experimentation was conducted with the approval of the Institutional Animal Care and Use Committee of the University of California, San Francisco, CA. Mice expressing the doxycycline-responsive reverse tetracycline transactivator (rtTA) driven by the rat Clara cell secretory protein (CCSP) promoter, mice with a tetracycline-responsive promoter element controlling expression of human MYC, and mice with a tetracycline-responsive promoter element controlling expression of human *N1ICD*, have been previously described [22]. Transgenic mice were fed with a supplemental diet containing doxycycline (200 mg/kg). Tissue sections were obtained and RNA samples were extracted as previously reported [22].

### Immunohistochemical analysis and scoring

Surgical specimens were collected by the Tissue Retrieval Services, and a tissue array comprising 50 lung ADC and 6 normal adjacent tissue (NAT) samples were prepared as 1 mm diameter cores in duplicate on two slides, totaling 4 cores per tissue, by the Histopathology and Imaging Shared Resource, at Rutgers Cancer Institute of New Jersey. Tumor/tissue quality was monitored under the guidance of a clinical pathologist. Immunohistochemical staining was done by permeabilizing with 0.1% Triton X-100 in PBS for 10 minutes, quenching endogenous peroxides with 3% hydrogen peroxide for 10 minutes, followed by blocking, as well as primary and secondary antibody incubation. Immunoreactivity was visualized with 3,3'-diaminobenzidine (DAB, Sigma). The primary antibodies used were: *Sox9* (1:50, HPA001758, Sigma), E-cadherin (pre-diluted, 760-4440, Ventana), Notch1 (1:100, EP1238Y, Epitomics).

Samples for which at least 25% of the tumor cells stained positive were considered positive. Staining intensity was scored as 0, negative; 1, weak; 2, moderate; and 3, strong. Scoring was performed in a blinded manner by two clinical pathologists and discordant results were discussed until a consensus was reached.

### Western blot analyses

Monolayer cells were washed and lysed with freshly prepared lysis buffer. Total cell lysates (20 μg) were assayed by western blot using standard procedures. Cleaved Notch1 and E-cadherin were visualized using high-intensity ECL. All others were visualized with standard ECL. Protein levels in all experiments were normalized to either *GAPDH* or α-tubulin. The following antibodies were used: *Sox9* (AB5535, Millipore), cleaved Notch1 (Val1744, 4147S, Cell Signaling), full-length Notch1 (C20, 6014, Santa Cruz), *Hes1* (25392, Santa Cruz), E-cadherin (21791, Santa Cruz), *GAPDH* (MAB374, Millipore) and α-tubulin (T9026, Sigma).

### Real-time quantitative RT-PCR (Q-PCR)

Total RNA was isolated using TRIzol (Invitrogen) and further purified using Qiashredder columns then by the RNeasy Mini kit (both from Qiagen Ltd., Germany) following standard protocols. RNA quantity and quality was assessed using a Nanodrop spectrophotometer. Reverse transcription was carried out using the Qiagen QuantiTect Reverse Transcription Kit in accordance with the manufacturer's instructions, followed by Q-PCR on a Stratagene Mx3005P machine using SYBR Green according to standard procedures. Primers were synthesized by IDT and results were analyzed using Microsoft Excel. Each assay was done at least in biological and PCR triplicate. Specificity of PCR amplification was assessed regularly using a melting-curve analysis. β-actin was used as the internal standard for normalization.

### siRNA and plasmid transfections

For siRNA transfection, a mixture of 20 pM siRNA (3 different siRNA sequences pooled) and 1 μL RNAiMax was diluted in Optimem. Experiments were performed 48- or 72-hours post-transfection. siRNA oligos were obtained from Santa Cruz (siNotch1- sc-36095; siNotch2- sc-40137; siNotch3- sc-37135; siNotch4- sc-40135; si*Sox9*- sc-36533; scr- sc-37007; FITC-A- sc-36869). Plasmid transfections were performed using Fugene transfection reagent. *N1ICD* cloned into pcDNA3.0 (Invitrogen) was a kind gift of Dr. Lucio Miele. The *SOX9*-pCMV-Tag2V expression vector was a kind gift of Dr. I-Shou Chang. In all experiments, scrambled siRNA sequence, or vector-only plasmids, or both, were used as controls.

### Chromatin immunoprecipitation assay

The chromatin immunoprecipitation (ChIP) assay was done according to the manufacturer's protocol (Upstate Biotechnology) in triplicate and on 2 separate occasions. In short, chromatin was cross-linked to DNA by adding formaldehyde to tissue culture dishes to a final concentration of 0.75%. Formaldehyde was neutralized and cells were scraped, washed, and resuspended in lysis buffer. Samples were sonicated to 200-500 bp fragments, diluted in ChIP dilution buffer, pre-cleared with salmon sperm DNA–protein A agarose slurry, centrifuged, and the supernatant was collected. Samples were incubated with immunoprecipitating Notch1 or control IgG antibody overnight at 4°C with rotation. Immune complexes were collected with salmon sperm DNA–protein A agarose slurry, then washed with low-salt immune complex buffer, high-salt immune complex buffer, LiCl immune complex buffer, and twice with TE buffer. Input DNA was removed for later normalization, then remaining samples were eluted, cross-links were reversed by incubation at 65°C overnight, and DNA was purified using the Qiagen Gel Extraction kit. Quantitative PCR was performed as outlined above, using the following primer to amplify the *Sox9* Site 4 (Fig. 4A) promoter region: forward primer: 5'- GGACTGCTGTGCTG-TGATTG-3', reverse primer: 5'- AGTTTCGAGCTCCGCTTTCG -3'. Samples were subsequently run on a 1.5% gel.

### Site directed mutagenesis and luciferase assay

The pGL2 firefly luciferase reporter plasmid under the control of the *Sox9* promoter was a kind gift of Dr. William L. Farrar. Mutations were induced using site-directed-mutagenesis primers in conjunction with the Agilent QuikChange II Site Directed Mutagenesis kit, according to the manufacturer's protocol. Cells were co-transfected with one of these plasmids and either the *N1ICD* or empty vector in triplicate. After 48 hrs, cells were harvested for determination of luciferase activity by a luminometer. A Renilla luciferase plasmid was used for normalization. Mutagenesis primers are listed in Supplementary Methods.

### Wound healing motility assay

Cells were plated such that they would be 95-100% confluent at the start of the assay. Cells were transfected 48 hours prior to creating a single scratch wound in triplicate wells. The wound closure experiment was performed in 2% fetal bovine serum to reduce potential differences in cell proliferation. Images were obtained at various time-points after wounding.

### Matrigel invasion assay

H1299 cells were transfected 48 hours prior to trypsinization and plated onto Matrigel invasion chambers (BD Biosciences) without serum. The chemoattractant in the lower chamber was 10% fetal bovine serum. The invasion assay was conducted for a minimum of 24 hours to reduce confounding due to potential differences in cell proliferation. Uninvaded cells were removed, and invaded cells were fixed with 4% PFA for 10 minutes, and stained with 0.05% crystal violet. Invaded cells were manually scored.

### Statistical analysis

All reported values represent the mean and standard deviation (SD) of at least three independent experiments, unless otherwise noted. All experiments were performed in biological triplicate and repeated at least once. Student's t-tests were used for statistical comparisons. Correlation between *Sox9* and E-cadherin staining was done by a Kruskal-Wallis test. Correlation coefficients between mRNA levels in microarray datasets were obtained by Pearson correlation. A P value ≤ .05 was considered statistically significant. All tests were done using Microsoft Excel or Stata version 12.

## SUPPLEMENTARY FIGURES AND METHODS


