# Supplementary material for: Sox9 mediates Notch1-induced mesenchymal features in lung adenocarcinoma
Source: Oncotarget. 2014 May 13;5(11):3636–50. doi: 10.18632/oncotarget.1970 (PMC4116509; doi:10.18632/oncotarget.1970)
Supplement: Supplementary file 1 [file oncotarget-05-3636-s001.pdf]

Sox9 mediates Notch1-induced mesenchymal features in lung adenocarcinoma

Supplementary Material

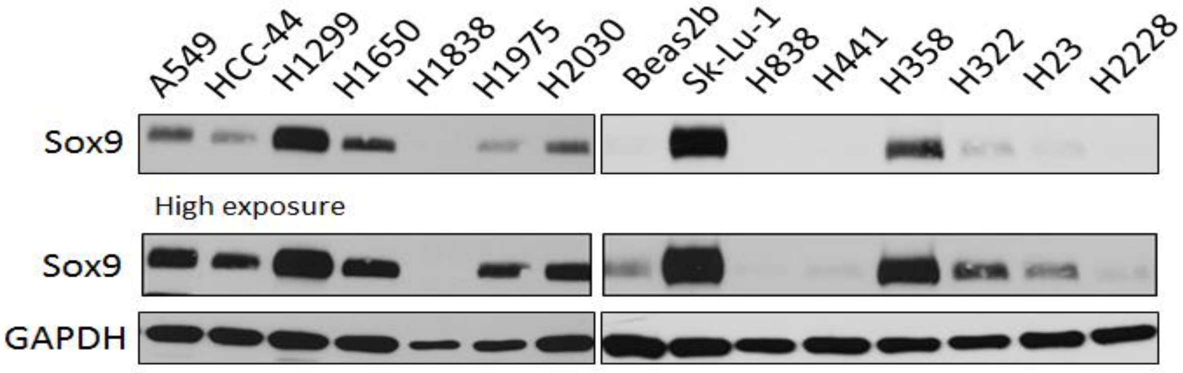

Supplementary Figure S1: *Sox9* protein expression in lung adenocarcinoma cell lines. A panel of 14 lung adenocarcinoma cell lines and 1 immortalized bronchial epithelial cell line (Beas2b) were analyzed for Sox9 protein levels by western blot. Protein expression levels of *GAPDH* are shown in the lower panels.

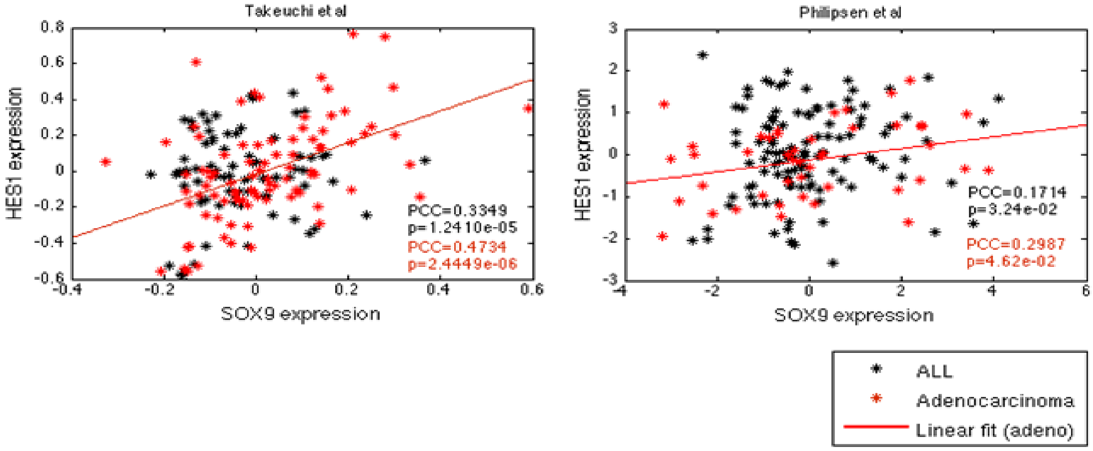

Supplementary Figure S2: Correlation between *SOX9* and *HES1* mRNA levels. Linear regression curves are shown for normalized *SOX9* and *HES1* mRNA levels (46, 62). Black asterisks indicate expression data for non-small cell lung cancer cases whereas red asterisks indicate expression levels within the lung ADC subgroups. Pearson correlation coefficients (PCC) and P values are shown.

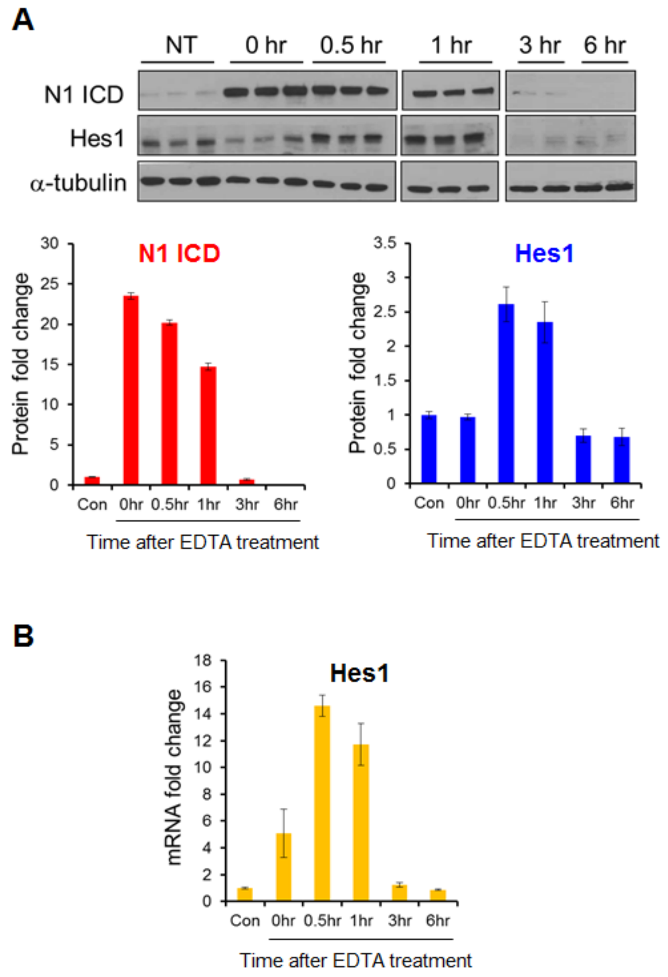

Supplementary Figure S3: EDTA induces Notch activation. Lung adenocarcinoma A549 cells were treated with 1 µM EDTA for 30 minutes and subjected to a "chase" period after EDTA treatment to monitor Notch activation kinetics. (A) Western blot analyses of Notch1 intracellular domain (*N1ICD*) and the canonical Notch1 target gene *Hes1* shows that Notch1 is activated immediately after EDTA treatment and induction of *Hes1* is maximal 0.5 - 1 hour-post EDTA treatment. Representative bands from multiple experiments are shown. In the lower panel, protein levels in triplicate experiments were quantified by densitometry, normalized to α-tubulin expression, and graphed as the mean fold change with standard deviation error bars. (B) Quantitative RT-PCR of *HES1*, normalized to the β-actin housekeeping gene shows that *HES1* mRNA was maximally upregulated 0.5 – 1 hour-post EDTA-induced Notch activation.

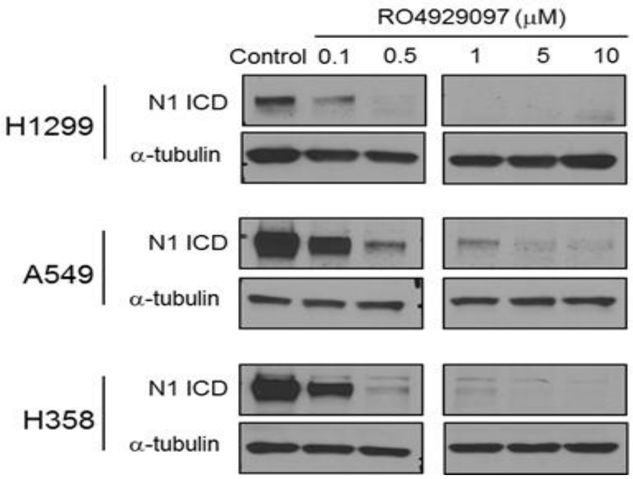

Supplementary Figure S4: Inhibition of Notch1 activation by the gamma secretase inhibitor RO4929097. Lung adenocarcinoma cell lines were exposed to the indicated concentrations of RO4929097 for 72 hours. Notch1 intracellular domain (N1 ICD) was probed by western blot to assess inhibition of Notch1, compared to the α-tubulin housekeeping gene. One μM RO4929097 was the optimal concentration used for inhibiting Notch1 activation across cell lines in the manuscript.

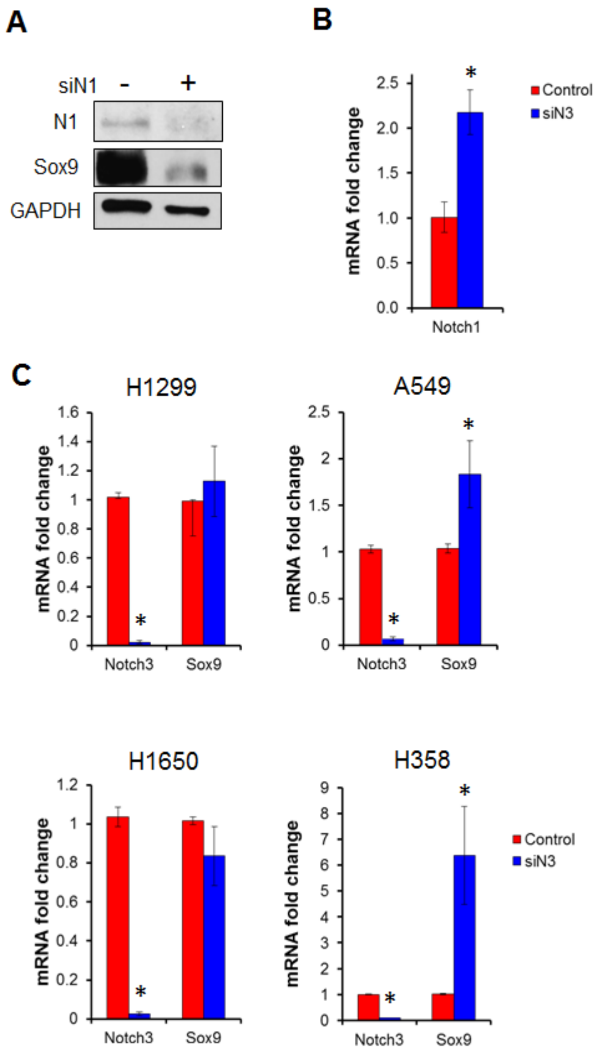

Supplementary Figure S5: Notch 1, but not Notch3 regulates *Sox9* expression in lung adenocarcinoma. (A) Western blot analyses of Notch1 and *Sox9* in H1299 cells demonstrates that transient knockdown of Notch1 by siRNA (siN1) decreases *Sox9* protein expression, compared to a scrambled siRNA sequence. (B) RT-PCR quantified mRNA levels of Notch1 are shown in A549 cells 48 hours after transient transfection with siRNA against Notch3 as compared to transfection with a scrambled siRNA (Control); *, $P < 0.05$. (C) Mean mRNA levels and standard deviations of Notch3 and *Sox9* are shown in lung ADC cells 48 hours after transient transfection with siRNA against Notch3 as compared to transfection with a scrambled siRNA (Control); *, $P < 0.05$.

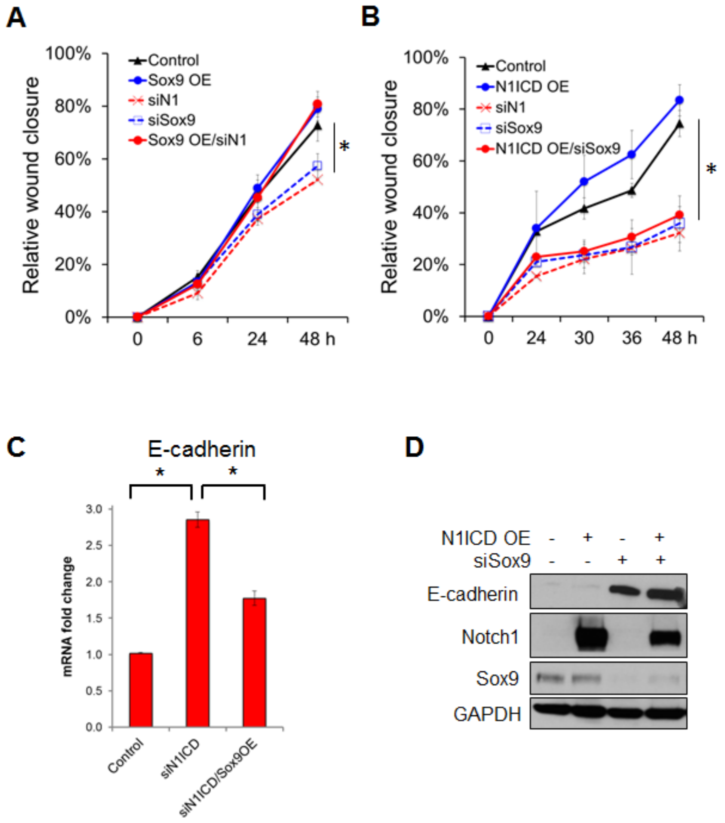

Supplementary Figure S6: *Sox9* mediates Notch1-induced cell motility and E-cadherin expression. (A) The migration ability of A549 cells was examined with a wound healing assay starting at 48 hours after transient knockdown of Notch1 or *Sox9*, or overexpression (OE) of *Sox9*. The control sample was transfected with both a scrambled siRNA sequence and empty vector. The mean migration distance of the wound edge, and standard error of the mean, is shown in all treatment compared to control cells; *, $P < 0.05$. (B) The migration ability of A549 cells was examined with a wound healing assay starting at 48 hours after transient knockdown of Notch1 or *Sox9*, or overexpression (OE) of *N1ICD*. The control sample was transfected with both a scrambled siRNA sequence and empty vector. The mean migration distance of the wound edge, and standard error of the mean, is shown in all treatment compared to control cells; *, $P < 0.05$. (C) Mean mRNA levels and standard deviations of E-cadherin expression in are shown for H1299 cells 72 hours after transient transfection with siRNA against Notch1 in combination with a vector control or a plasmid overexpressing Sox9, as compared to transfection with a scrambled siRNA (Control); *, $P < 0.05$. (D) Notch1, E-cadherin and *Sox9* were analyzed by western blot 72 hours after transient overexpression of activated Notch1 (N1ICD OE), knockdown of *Sox9*, or transfection with a scrambled siRNA sequence and empty vector, in A549 cells.

Supplementary Methods

The following primers were used for quantitative PCR:
Notch1 (human)
forward: 5′-TGGACCAGATTGGGGAGTTC-3′,
reverse: 5′-GCACACTCGTCTGTGTTAC-3′;
Notch1 (mouse)
forward: 5′-CTACTCACGCTCTGATGCCG-3′,
reverse: 5′-TCTGGAAGACACCTTGTGCG-3′;
Sox9 (human)
forward: 5′- AGCGAACGCACATCAAGAC-3′,
reverse: 5′-CTGTAGGCGATCTGTTGGGG-3′;
Sox9 (mouse)
forward: 5′-AGCGAACGCACATCAAGAC-3′,
reverse: 5′- CTGTAGGAGATCTGTTGGGG-3′;
Hes1
forward: 5′-TCAACA-CGACACCGGATAAA-3′,
reverse: 5′-CCGCGAGCTATCTTTCTTCA-3′;
Hes 5
forward: 5′-TCAGCCCCAAAGAGAAAAAC-3′,
reverse: 5′-TAGTCCTGGT-GCAGGCTCTT-3′;
Hey1
forward: 5′- ATCTGCTAAGCTAGAAAAAGCCG-3′,
reverse: 5′-CGTCAAAGTAACCTTTCCCTCCT-3′;
Notch3
forward: 5’- CGTGGCTTCTTTCTACTGTGC-3′,
reverse: 5′-CGTTCACCGGATTTGTGTCAC-3′;
E-cadherin
forward: 5′-GGTCACAGCCACAGACGCGG-3′,
reverse: 5′- GGAAACTCTCTCGGTCCAGCCCA-3′;
β-actin
forward: 5′- AGGCACCAGGGC-GTGAT-3′,
reverse: 5′-GCCCACATAGGAATCCTTCTGAC-3′.

The following primers were used for site-directed mutagenesis:
Site 1:
forward:
5′GAGAGAGCAGTGAAAAGAAATGTCGGAGAGTACTGTAGATCCTAGTCTAGACACACACACTT-3′,
reverse: 5′AAGTGTGTGTGTCTAGACTAGGATCTACAGTACTCTCCGACATTTCTTTTCACTGCTCTCTC-3′;
Site 2:
forward: 5′-AATGCCAGAATTTTAGTGCCTTCTCCTGATTCATCTTGTGTGTGTGTGTGTGT-GTGTGCG -3′,
reverse: 5′-CGCACACACACACACACACACACAAGATGAATCAGGAGAAGGCACTAAAA-TTCTGGCATT -3′;
Site 3:
forward: 5′- TCCAATCAGCTGC-CTGCCAACCTGTATGCTGCTGTGCTGTGATTGGCGGG -3′, reverse: 5′-CCCGCCAATCACAGCACAGCAGCATACAGGTTGGCAGGCAGCTGATTGGA -3′;
Site 4:
forward: 5′- TCGAGCTCCGCTTTCGGCTCTCCAACGATACACCCAGGGTCTCTTTAATAAAT-ACTCC -3′,
reverse: 5′- GGAGTATTTATTAAAGAGACCCTGGGTGTATCGTTGGAGAGCCGAAAGCGGA-GCTCGA -3′;
Site 5:
forward: 5′- GCGGAGCTCGAAACTGACTGGAAACTTCAAGTATGCGGAGACTCGCCAG -3′, reverse: 5′-CTGGCGAGTCTCCGCATACTTGAAGTTTCCAGTCAGTTTC-GAGCTCCGC -3′.
